# Solid-Phase Synthesis of Cellulose Acetate Butyrate as Microsphere Wall Materials for Sustained Release of Emamectin Benzoate

**DOI:** 10.3390/polym10121381

**Published:** 2018-12-13

**Authors:** Aimin Huang, Xuanhai Li, Xingtang Liang, Yanjuan Zhang, Huayu Hu, Yanzhen Yin, Zuqiang Huang

**Affiliations:** 1 School of Chemistry and Chemical Engineering, Guangxi University, Nanning 530004, China; ham2003cn@163.com (A.H.); xuanhli@gxu.edu.cn (X.L.); zhangyj@gxu.edu.cn (Y.Z.); yuhuahu@163.com (H.H.); 2 Qinzhou Key Laboratory of Biowaste Resources for Selenium-Enriched Functional Utilization, College of Petroleum and Chemical Engineering, Qinzhou University, Qinzhou 535011, China; yinyanzhen2018@163.com; 3 Medical College of Guangxi University, Nanning 530004, China

**Keywords:** cellulose acetate butyrate, solid-phase synthesis, mechanical activation, microspheres, emamectin benzoate, release

## Abstract

Emamectin benzoate (EB), a widely used pesticide, is prone to decomposition by ultraviolet light and suffers from the corresponding loss of efficacy. The timed release of EB based on microspheres is one of the effective methods to solve this issue. As a non-toxic cellulose ester, cellulose acetate butyrate (CAB) is regarded as one of the best wall-forming materials for microcapsules with a good controlled release performance. Herein, two methods—mechanical activation (MA) technology and a conventional liquid phase (LP) method—were employed to synthesize different CABs, namely CAB-MA and CAB-LP, respectively. The molecular structure, rheological property, and thermal stability of these CABs were investigated. The two CABs were used to prepare microspheres for the loading and release of EB via an o/w (oil-in-water) solvent evaporation method. Moreover, the performances such as drug loading, drug entrapment, and anti-photolysis of the drug for these microspheres were studied. The results showed that both CABs were available as wall materials for loading and releasing EB. Compared with CAB-LP, CAB-MA presented a lower molecular weight and a narrower molecular weight distribution. Moreover, the MA method endowed the CAB with more ester substituent groups and less crystalline structure in comparison to the LP method, which had benefits including pelletizing and drug loading.

## 1. Introduction

Due to advantages such as high efficiency, low toxicity, low residue, and low pollution, emamectin benzoate (EB) has been widely used as a biopesticide through the forms of emulsion, microemulsion, suspension, and granules dispersed in water [1]. However, EB can be readily degraded by ultraviolet light, which degrades its efficacy and limits the application of EB [2]. The development of the timed release of EB by embedding it into microcapsules or microspheres composed of polymers is one of the promising methods to solve this issue. Generally, at the initial stage of the utilization, drugs distributed on the surface of microspheres are directly diffused into the environment, resulting in a fast release at the beginning stage. Then, the active ingredient that is incorporated into the carrier is released slowly via the inner channels or the decomposition of the microsphere component [3]. This release mechanism presents several advantages in the application of pesticide. First, the slow release of effective components wrapped in the polymer carrier prolongs the period of validity and improves the utilization rate. Meanwhile, the contact toxicity and inhalation toxicity of toxic pesticides toward crops, beneficial insects, and human beings will be reduced. Furthermore, the internal effective components will be protected by the carrier materials, which decrease the decomposition of drugs by environmental factors including light and microorganisms, and therefore boost its utilization [2]. For the wall materials, those with biocompatibility and biodegradation such as chitosan, starch, and polylactic acid have attracted widespread attention [4,5,6]. As the most abundant green renewable resource, cellulose is a promising candidate as a wall-forming material for microspheres. However, highly crystalline cellulose has poor solubility, which limits its applicability [7]. The cellulose ester, a cellulose derivative prepared via the esterification of cellulose, has advantages including high solubility, good film-forming ability, high glass-transition temperature, and biodegradability. Due to such advantages, it has attracted more and more attention as a microcapsule material [8]. Cellulose acetate butyrate (CAB) is a typical cellulose ester that is prepared by the simultaneous esterification with acetyl and butyryl groups [9]. With excellent UV resistance, good compatibility, and good film-forming ability, CAB has been regarded as one of the most widely used cellulose esters for microsphere wall materials [10,11]. Generally, the drug encapsulation and controlled release of CAB microcapsules is affected by the physicochemical properties of a polymer such as its crystalline structure and viscosity, which is determined by the degree of substitution and molecular weight [12]. The CAB with low viscosity would be favorable for the pelletization and encapsulation of drugs. However, the physicochemical properties of CAB are significantly influenced by the preparation methodology [12].

Recently, our research group has developed a new green method for preparing cellulose derivatives by mechanical activation-assisted solid-phase reaction (MASPR) technology without using organic co-reagents and solvents [13,14,15,16]. In the MASPR process, the mechanical activation (MA) fractures the crystalline structure of cellulose to decrease the particle size and make a new surface with highly active hydroxyl groups. Meanwhile, the MA promotes the penetration of other reactants into the inside of the cellulose. All of these significantly enhance the efficiency of cellulose modification. Furthermore, without any use of additional solvents, this process simplifies the operation and fully exploits the mechanical energy for the reaction. 

Based on these considerations, in the current work, we prepared CAB by the MA technology and the conventional liquid phase (LP) method for microcapsule materials. The physicochemical properties such as the molecular structure, crystalline structure, and rheological property for the two kinds of CAB (CAB-MA and CAB-LP) were characterized to study their differences. Then, these two types of CABs were used as wall materials to prepare CAB microspheres containing emamectin benzoate (EB/CAB microspheres) through the solvent evaporation method. The drug loading, drug release, morphology, and the photodegradation of the EB/CAB microspheres were systematically studied. This work provided a promising method to prepare CAB as wall materials of microsphere for the slow release of EB.

## 2. Materials and Methods 

### 2.1. Materials

Microcrystalline cellulose (MCC) with a degree of polymerization of about 200 was obtained from Shanghai Yuanye Bio-Technology Co., Ltd. (Shanghai, China). Acetic anhydride, butyric acid, acetone, dichloromethane, absolute alcohol, methyl alcohol, and acetonitrile were supplied by Aladdin Industrial Co., Ltd. (Shanghai, China). Acetonitrile was chromatographically pure, and all of the other reagents were of analytical grade and used without further purification.

### 2.2. Preparation of CAB-MA and CAB-LP

CAB-MA prepared by the MA method was performed in a customized stirring ball mill [17]. Typically, 10.0 g of MCC mixed with butyric acid, acetic anhydride, and sulfuric acid catalyst (molar ratio of anhydroglucose unit (AGU) of MCC:butyric acid:acetic anhydride:sulfuric acid = 1:6:3:0.06) was poured into a 1200-mL jacketed stainless steel chamber containing 500 mL of zirconium oxide milling balls (Φ = 5 mm). The mixture was subjected to the milling at 350 rpm for one hour at a temperature of 75 °C, which was controlled by a circulation of thermostatic water in the chamber jacket. The esterification reaction was simultaneously conducted in the chamber. Then, the resulting sample was separated from the milling balls and treated by the washing-filtration process with water and absolute alcohol, respectively. This process was repeated for several cycles until the filtrate was neutral to remove unreacted reagents. The filter cake was collected and vacuum dried at 55 °C for two days to obtain the CAB-MA. 

CAB-LP was prepared according to the literature with some modification [18]. Briefly, 10.0 g of MCC was firstly pretreated via immersion in a 120-mL mixture of acetic acid and acetic anhydride with the volume ratio of 5:1 for 12 h at ambient temperature. After filtration, the pretreated MCC, butyric acid, acetic anhydride, and sulfuric acid catalyst with the same molar ratio to the above-mentioned method were magnetically stirred for one hour at 75 °C for esterification. After washing and drying in a similar manner as described above, the CAB-LP was acquired.

### 2.3. Characterizations of CAB

The ^13^C nuclear magnetic resonance (NMR) spectra of CABs were recorded on a Bruker Advance HD III 600 MHz spectrometer (Flanders, Switzerland) at room temperature, by using deuterated chloroform (CDCl_3_) and tetramethylsilane (TMS) as the solvent and internal standard, respectively. Degree of substitution (DS) values for CABs were determined according to Tezuka and Tsuchiya’s method based on the ^13^C NMR spectra of cellulose esters [19]. X-ray diffraction (XRD) experiments for MCC and CABs were conducted on an Ultima IV diffractometer (Tokyo, Japan) with a Cu Kα X-ray beam at 40 kV and 24 mA. The relative molecular weight of CAB was determined by a LC-20AT high-performance liquid gel chromatograph (SHIMAZU, Japan) with Shodex GPC KF-804L C004091 HPLC column at 25 °C. Polystyrene and tetrahydrofuran were used as the molecular weight marker and mobile phase, respectively. The rheological properties of CAB acetone solutions were measured on an Anton Paar MCR 302 dynamic rotational rheometer (Graz, Austria), using the Rheo Plus software. All of the measurements were carried out in the CC27 concentric cylinder geometry. Thermogravimetric analyses were executed on a TGA Q50 thermogravimetric analyzer (Wilmington, DE, USA).

### 2.4. Preparation of EB/CAB Microspheres

EB/CAB microcapsules were prepared with an o/w solvent evaporation method within an open cylindrical reactor (h = 155 mm, d = 112 mm) combined with a high-power thermostatic magnetic stirrer. Typically, 200 mg of CAB and 100 mg of EB were added into five mL of chloroform, and then stirred for two minutes. Afterward, the obtained viscous liquid was added dropwise into the polyvinyl alcohol solution (300 mL, 1% *w*/*v* and 88% hydrolyzed) under an 800-rpm stirring. This mixture was continuously stirred for 20 min at room temperature. After that, the temperature of the system was raised up to 55 °C and another one hour of stirring (500 rpm) was conducted. Finally, the EB/CAB complex microspheres were obtained after vacuum filtration, washed with deionized water, and vacuum dried at 50 °C. Blank CAB microspheres were also attained by the same method without adding EB.

### 2.5. Characterizations of EB/CAB Microspheres

The particle size and size distribution of EB/CAB microspheres were determined using a BT-9300st laser particle size distribution instrument (Dandong, China). Morphologies for EB/CAB microspheres were observed in an S-3400N scanning electron microscope (SEM, HITACHI, Tokyo, Japan).

### 2.6. Characterizations of the Drug Loading of EB/CAB Microspheres

First, 40 mg of EB/CAB microspheres were immersed in 100 mL of methyl alcohol, and then ultrasonically treated for 20 min to completely discharge the embedded EB from the microspheres. The EB concentration of the methyl alcohol solution was measured by a TCC-300 CD high-performance liquid chromatography (HPLC) system equipped with a C18 column (Waters Sunfire). A mixture of methanol and acetonitrile with a volume ratio = 55:45 was used as the mobile phase. The mass fraction of EB in EB/CAB microspheres, drug-loading rate, and entrapment rate were calculated according to the literature [20].

### 2.7. Release Studies of EB from EB/CAB Microspheres

EB release tests from the microspheres were conducted in 0.05 M of phosphate buffer solution (PBS) (PH 7.0, 20% methanol) by the membrane diffusion method. First, 150 mg of EB/CAB microspheres were placed in a dialysis bag and put into a conical flask containing 120 mL of PBS. The conical flask was placed in an oscillator with 25 °C and subjected to a continuous shaking at 120 rpm. Then, two mL of release medium were withdrawn at 24-h intervals for determining the EB content. The changes of the morphology of CAB microspheres during the release process were recorded by an optical microscope.

### 2.8. Experiments for EB Photodegradation

The photodegradation of EB was conducted under sunlight. Briefly, about 50.0 mg of EB/CAB microspheres were placed in a Petri dish containing 10 mL of water and exposed to sunlight for photodegradation. For the control sample, the same system containing 50.0 mg of EB/CAB and 10 mL of water was placed in a dark room. After maintaining for one hour, two hours, four hours, eight hours and 10 hours, both samples, including the exposed one and the unexposed one, were treated by ultrasonication in methanol to completely release EB. Then, the content of EB was detected. The content of EB for the exposed EB/CAB was labeled as *m*_e_, while the control one was *m*_c_. The photodegradation rate (*P*_r_) of EB was calculated according to the equation (*P*_r_ = 100% (*m*_c_ − *m*_e_)/*m*_c_). The EB was also conducted in a similar manner as the above-mentioned process for determining the *P*_r_.

## 3. Results and Discussion

### 3.1. Distribution of Substituted Groups in CABs with Different Synthesis Methods

**Figure 1:**
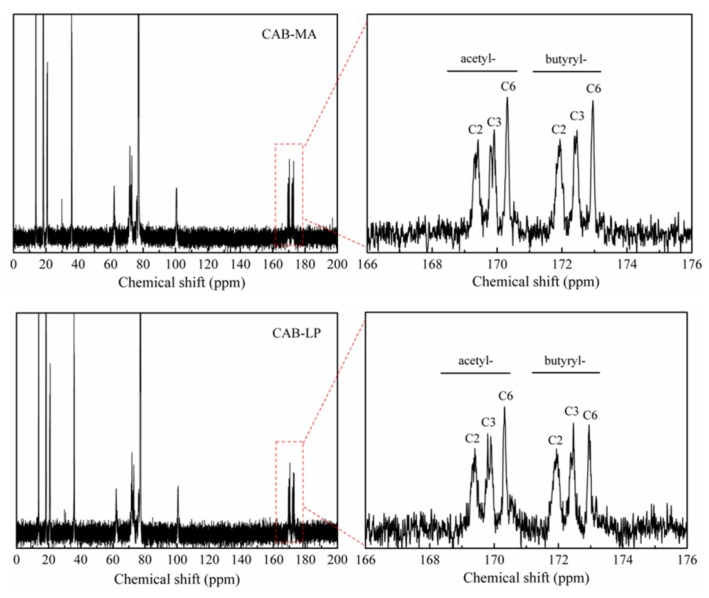
^13^C NMR spectra of cellulose acetate butyrate by mechanical activation (CAB-MA) and liquid phase methods (CAB-LP).

The ^13^C spectra of CAB-MA and CAB-LP are shown in Figure 1. Two triplets at the range of 169–174 ppm, assigned to the acetyl carbonyl and the butyryl carbonyl, respectively, appeared in the spectra of both CABs, indicating the successful preparation of cellulose mixed esters via both approaches [21]. For the acetyl carbonyl triplet, the peaks at 169.3 ppm, 169.8 ppm, and 170.3 ppm are attributed to the carbonyl signal of the acetate group in the two, three, and six positions of the AGU. While the peaks at 171.9 ppm, 172.4 ppm, and 172.9 ppm in the butyryl carbonyl triplet are assigned to the carbonyl signal of the butyrate-substituted group in the two, three, and six positions. According to Tezuka and Tsuchiya’s method, the DS values for every position and the total DS were calculated and listed in Table 1. During the process of the MA method, all of the hydroxyl groups in the cellulose were activated by ball milling to react with the esterifying agents [18]. The primary hydroxyl of C6 at the AGU, with a higher activity in comparison to the secondary hydroxyl at C3 and C2, resulted in a significantly greater degree of substitution at C6 than at C3 and C2. However, in the LP process, the substituent in C6 was close to that of C3 and C2, namely, the esterification of AGU by the LP process was more homogeneous than that of the MA method. This would be due to the solvation effect of the acetic acid and acetic anhydride in the pretreatment process, which protected the C6-OH group [22]. In addition, the total DS of CAB-MA (DS_total_ = 2.73) was greater than that of CAB-LP (DS_total_ = 2.53), indicating the advantages of the MA method for preparing CAB.

### 3.2. Effect of Synthesis Method on Crystal Structure of CAB

As wall materials, the crystallinity directly affects its mechanical strength and sealing property [23]. The higher the degree of crystallinity, the smaller the distance between molecules, which increases the intermolecular cohesive force, and therefore increases the rigidity of the polymer chains. At the same time, the molecular chain within the polymer with a high degree of crystallinity will be bound to a certain extent, which hinders the movement of the molecular chain segment and results in a fragile microcapsule. Moreover, the crystal components are of high density and low porosity, which obstructs the diffusion of the drug from the inside of the microcapsule to the external environment [24]. Conversely, the polymer with low crystallinity promotes the movement of the molecular chains and enhances the flexibility. Therefore, these microcapsules prepared with a low crystallinity polymer exhibit a suitable release of drug [25].

**Figure 2:**
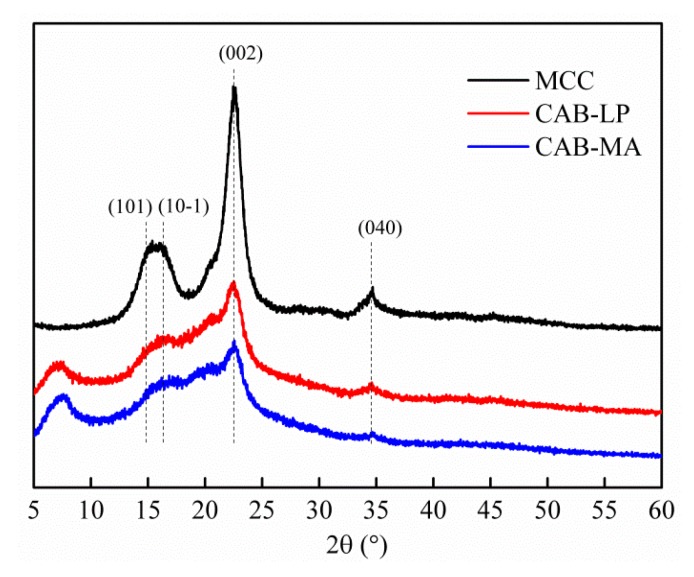
Typical XRD patterns of microcrystalline cellulose (MCC), CAB-LP, and CAB-MA.

The XRD patterns of MCC, CAB-MA, and CAB-LP are shown in Figure 2. The diffraction pattern of MCC revealed the crystalline planes of (101), (10-1), (002), and (040) at about 14.9°, 16.3°, 22.5°, and 34.8°, respectively, confirming the form of native cellulose I [26]. After an esterification reaction, the intensity of the relevant peaks decreased significantly, indicating that this esterification modification notably destroyed the crystal structure. The crystallinity indices (CrI, %) and the crystallite sizes (D_002_, nm) for these samples were calculated according to equations (1) and (2), respectively [27].

CrI = 100 (*I*_002_ − *I*_am_)/*I*_am_ (1)

D_002_ = *k λ*⁄(*β* cos*θ*) (2)

where, *I*_002_ and *I*_am_ are the intensity of the (002) plane and the diffraction intensity of amorphous region at 18.0°, respectively; and *k*, *λ*, *β*, and *θ* represent the Scherrer constant (0.94), the wavelength of X-ray (0.154 nm), the full-width at half-maximum (002) peak, and the diffraction angle of the (002) plane, respectively. As shown in Table 2, the CrI for CAB-MA is less than that of CAB-LP, while the D_002_ for CAB-MA is greater than that of CAB-LP, which is close to the D_002_ of MCC. These results would be attributed to the esterification reaction in the LP process occurring in the amorphous region and on the surface of the crystalline zone, which gradually destroys the crystal structure, but without influencing the crystallite size. However, in the MA process, MA not only induced the esterification reaction, but also broke and refined the cellulose particles, which enlarged the interplanar spacing.

### 3.3. Effect of Synthesis Method on Molecular Structure of CAB

**Figure 3:**
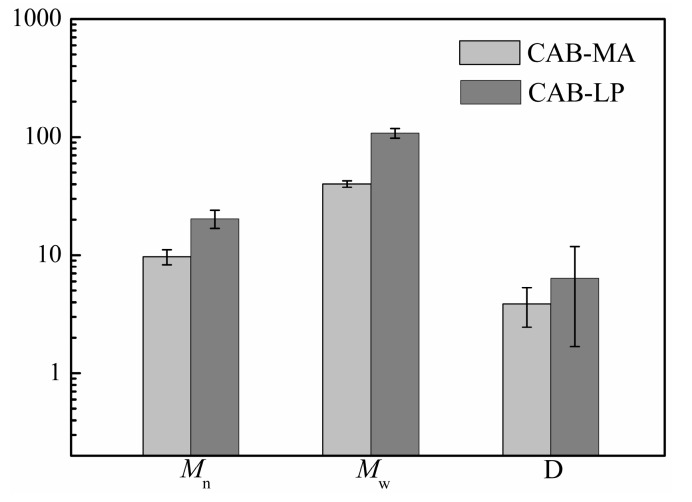
Relative molecular weight and molecular weight distribution of CAB-MA and CAB-LP (*n* = 3).

**Figure 4:**
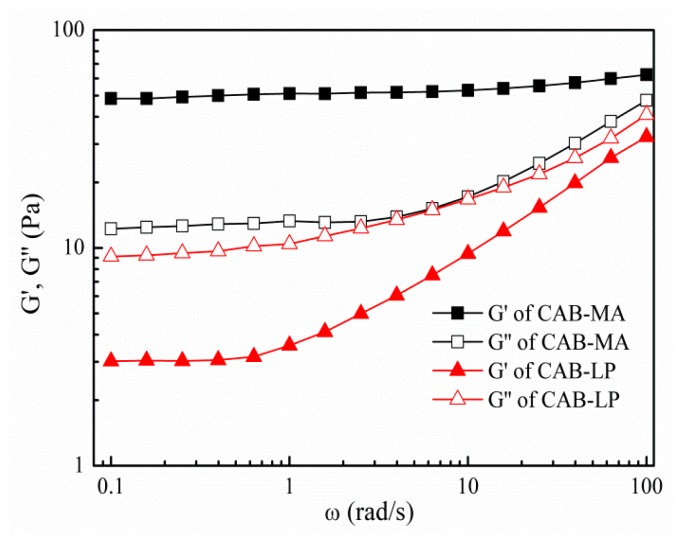
The dynamic frequency scanning curves of the CAB-MA and CAB-LP.

The number-average molecular weight (*M*_n_), weight average molecular weight (*M*_w_), and molecular weight distribution (D) for both CAB-MA and CAB-LP were determined by the gel permeation chromatography (GPC) method. As shown in Figure 3, the *M*_n_, *M*_w_, and D of CAB-LP are significantly greater than that of CAB-MA, indicating that the cellulose molecules and the CAB molecules are fractured under the repeated actions such as shear, impact, friction, and impact during the process of mechanical activation. With a low molecular weight and a narrow molecular weight distribution width, CAB-MA would be more soluble and dispersive than CAB-LP. This was proved by the rheological experiment, as shown in Figure 4. The energy storage modulus (G′) and the loss modulus (G″) are used to characterize the elastic properties and the viscosity of a polymer solution, respectively. The G′ for CAB-MA was greater than the G″ in the studied frequency range, indicating the characteristic of solid elasticity. However, the G′ for CAB-LP was smaller than the G″ in the same frequency range, confirming the typical liquid viscosity behavior. These results showed that the CAB-MA, with a greater DS, a lower molecular weight, and a narrower molecular weight distribution in comparison to the CAB-LP exhibited a stronger tendency to assemble. This is suitable as a wall material with good flexibility and mechanical strength for the efficient encapsulation of drug [28].

**Figure 5:**
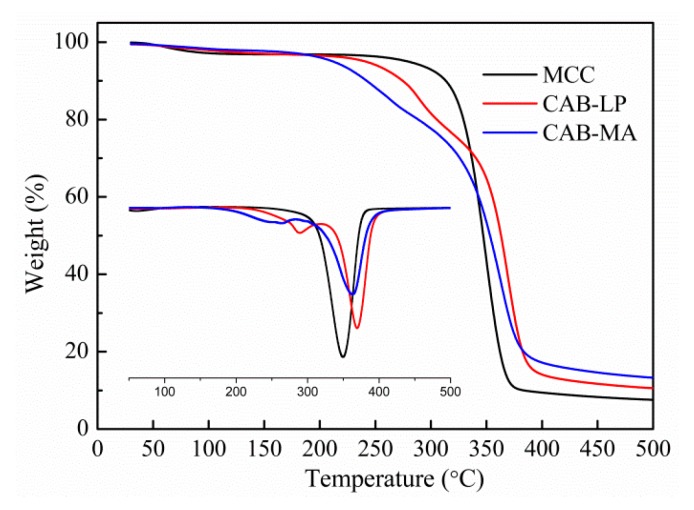
Thermogravimetric analysis (TGA) and derivative thermogravimetry (DTG) curves of MCC and CAB.

The thermogravimetric analysis (TGA) and derivative thermogravimetry (DTG) curves for MCC, CAB-MA, and CAB-LP are shown in Figure 5. Generally, the substituted ester groups, especially the groups on the highly active C6, are firstly decomposed in the process of thermal decomposition [29]. In the step of the decomposition of ester groups, the CAB-MA revealed an initial decomposition temperature and a DTG peak temperature at 189 °C and 263 °C, respectively, which were lower than that of CAB-LP (210 and 284 °C, respectively). These results are in consonance with the above-mentioned ^13^C NMR analysis, further confirming that MA is efficient technology to activate the cellulose for an esterification reaction with butyric acid and acetic anhydride.

### 3.4. Morphology of EB/CAB Microspheres

**Figure 6:**
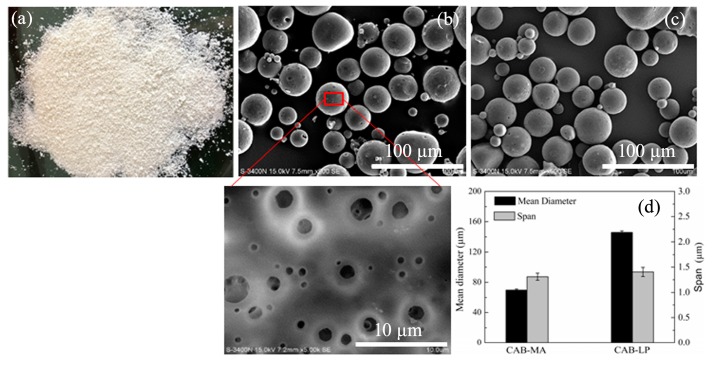
The photograph of the CAB microspheres containing emamectin benzoate (EB/CAB) (**a**); the SEM mages of microspheres prepared by CAB-MA (**b**) and CAB-LP (**c**), respectively; the mean diameter of EB/CAB-MA and EB/CAB-LP (**d**).

The CAB, with two kinds of substituted ester groups, presenting the hydrophobicity, is considered to be a favorable drug carrier of hydrophobic drugs such as EB [30]. In this study, EB was successfully entrapped into the CAB polymer matrix via the solvent evaporation method. The prepared EB/CAB microspheres are faint yellow, and present a good dispersion (Figure 6a). The microspheres prepared by both CAB-MA and CAB-LP showed a similar morphology, with numerous pores on their surfaces (Figure 6b,c). This porous structure would facilitate the infiltration of water to swell or disintegrate the microspheres in the application, which results in the release of the encapsulated drug [31]. Due to a lower molecular weight and a higher DS, CAB-MA had stronger association effect in comparison to CAB-LP, and revealed microspheres with smaller diameters, as determined by the laser particle size distribution instrument (Figure 6d).

### 3.5. Effect of Synthesis Method on Entrapment Performance of CAB

**Figure 7:**
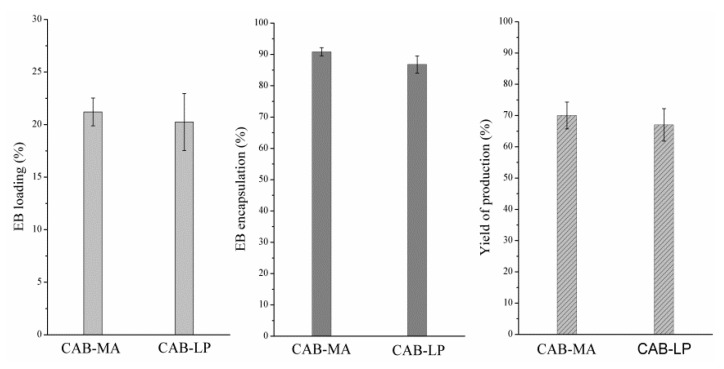
The results of emamectin benzoate (EB) loading, entrapment rate, and yield of production (*n* = 3).

In order to study the entrapment performance of CABs prepared by different methods, the EB/CAB-MA and EB/CAB-LP microspheres were produced under the same conditions. The EB loading (%), the EB entrapment rate (%), and the yield of production (%) for both microspheres are shown in Figure 7. Obviously, these entrapped characteristics of the microcapsules prepared by CAB-MA are greater than that of the microcapsules produced by CAB-LP. Usually, the entrapped characteristics are influenced by the molecular structures of the polymer matrix such as crystallization, molecular weight, and substituted groups [32,33]. The polymer matrix with a lower degree of crystallinity can improve the miscibility between the drug and the matrix, which induces a higher drug loading, entrapped rate, and yield of production in comparison to the polymer with a higher crystallinity. The polymer with a smaller molecular weight and more functional groups that are related to the drug molecular can stabilize the dispersion of a polymer and drug mixture. These also promote the encapsulation performance. Herein, the CAB prepared by MA technology presented a lower degree of crystallinity, a smaller molecular weight, and a higher content of substituted groups in comparison to the CAB produced by the LP method. Therefore, the microcapsules derived from CAB-MA exhibited a better encapsulation performance in comparison to the CAB-LP microspheres.

### 3.6. EB Release from Microspheres and the Anti-Photodegradation Effect

**Figure 8:**
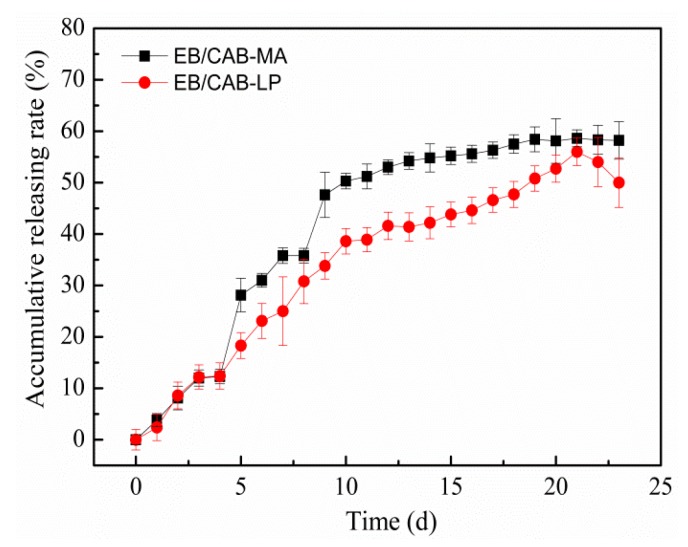
Releasing rate of EB from EB/CAB-MA and EB/CAB-LP microspheres (*n* = 3).

**Figure 9:**
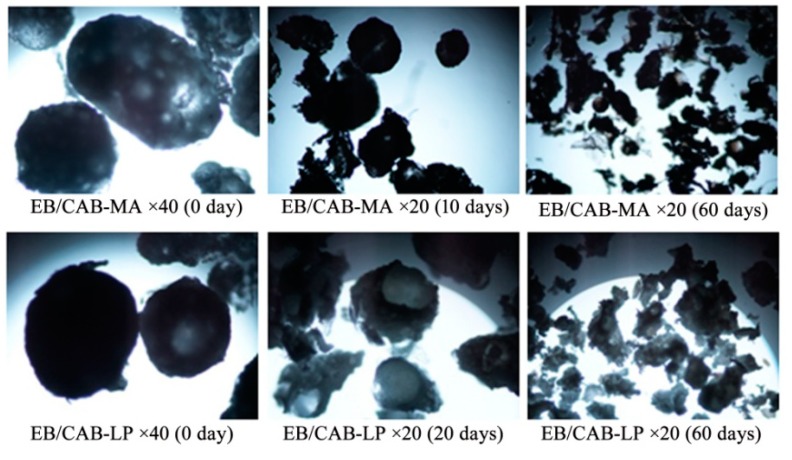
The optical photographs of EB/CAB-MA and EB/CAB-LP microspheres during the degradation process.

Figure 8 shows the release profiles of EB from the EB/CAB-MA microspheres and the EB/CAB-LP microspheres. At the initial stage (10 days), both microspheres displayed a fast release rate of EB, and 30–40% of EB was released in this period. After that, these microspheres exhibited a slow release rate. At the first step, the EB on the surface or outer layer of the microspheres would be readily discharged, and resulted in a fast release. The EB within the inside of the microspheres was entangled tightly by the CAB molecular, which prevented the release of EB until the fracture of the microsphere to produce a new surface. Therefore, the release was slow in this stage. After 20 days, the accumulative release of EB from microspheres was less than 60%. The slow release would prolong the useful life of EB. The structural changes exhibited by the microspheres can be seen in Figure 9. The hydrophilic polyvinyl alcohol, which is a low-content component of EB/CAB microspheres, would be swollen when the microspheres were immersed in the aqueous solution. The swelling of the polyvinyl alcohol would produce a force to rupture the microspheres [34]. Taken together, we could conclude that the release of EB from EB/CAB microspheres followed a self-propelled drug release mechanism [34]. Due to the smaller size, the EB/CAB-MA microspheres with a shorter diffusion path and larger diffusion surface showed a faster release of EB in comparison to the EB/CAB-LP microspheres. To study the kinetics of EB release from the EB/CAB microspheres, various kinetic models, including zero-order (Equation (3)), first-order (Equation (4)), and Higuchi square root (Equation (5)) were employed to fit the release results [35].

*Q*_t_/*Q*_0_ = *k t* (3)

(1 − *Q*_t_/*Q*_0_) = *k t* (4)

*Q*_t_/*Q*_0_ = *k t*^0.5^ (5)

where *Q*_t_ and *Q*_0_ are the amount of EB released at time *t* and the initial amount of EB in the microspheres, respectively; and *k* represents the corresponding rate constant. The correlation coefficient (*R*) values for the fitting were listed in Table 3. With a greater *R* value, the first-order kinetic model was more consistent with the release of EB from both kinds of microspheres in comparison to the other kinetic models.

**Figure 10:**
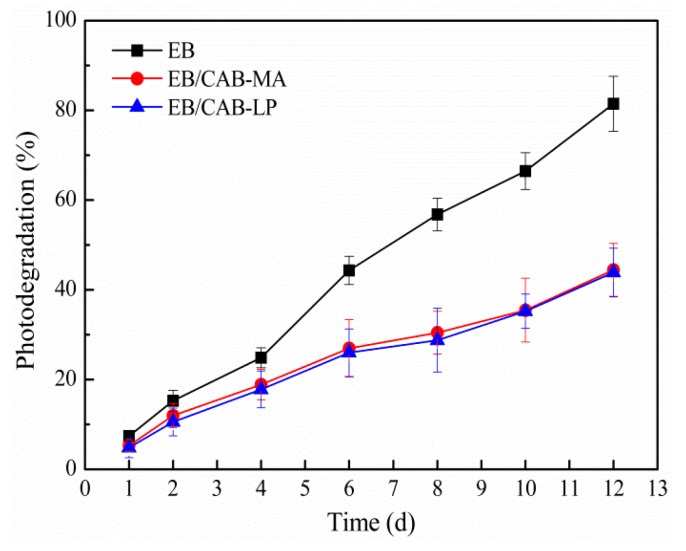
The photodegradation of EB in the control and entrapped in the microspheres (*n* = 3).

Figure 10 shows the photodegradation of EB in the control and entrapped in both types of microspheres. After exposure to sunlight for 12 days, more than 80% of the control EB was degraded. However, the EB that was entrapped in both microspheres exhibited a similar degradation rate, and only about 40% of the EB was decomposed under the same exposure time. This result indicates that CAB can encapsulate EB to protect it from the degradation by sunshine.

## 4. Conclusions

Cellulose acetate butyrate microspheres containing emamectin benzoate were prepared to promote the efficacy of emamectin benzoate by enabling slow release and providing resistance against photolysis. Following the self-propelled release mechanism, less than 60% of the loading emamectin benzoate was released from the microspheres during the first 20 days. Compared to the cellulose acetate butyrate prepared by the conventional liquid phase method, in which a pretreatment by the mixture of acetic acid and acetic anhydride was needed, the cellulose acetate butyrate produced by the mechanical activation-assisted technology showed a lower crystallinity, a smaller molecular weight, and a higher content of substituted groups. These benefited the pelletization of cellulose acetate butyrate, and improved the combination of polymer molecular and drug molecular, which therefore increased the encapsulation of emamectin benzoate. Moreover, the mechanical activation-assisted technology, without any use of additional solvents, was a highly efficient, easy-handling, and eco-friendly method for producing cellulose acetate butyrate. 

## Figures and Tables

**Table 1:** The distribution of substituted groups in the anhydroglucose unit (AGU) of CAB-MA and CAB-LP. DS: degree of substitution.

	DS of Butyryl				DS of Acetyl				DS_total_
	C_6_	C_3_	C_2_	DS_b_	C_6_	C_3_	C_2_	DS_a_	
CAB-LP	0.57	0.59	0.44	1.62	0.37	0.29	0.24	0.91	2.53
CAB-MA	0.69	0.52	0.47	1.67	0.43	0.33	0.29	1.05	2.73

**Table 2:** Values of CrI and *D*_002_ for MCC, CAB-LP, and CAB-MA (*n* = 3).

	MCC	CAB-LP	CAB-MA
CrI (%)	94.9 ± 1.9	44.7 ± 2.8	21.7 ± 5.2
*D*_002_ (nm)	3.5 ± 0.4	3.7 ± 0.1	4.2 ± 0.4

**Table 3:** *R* values of all models for releasing EB from EB/CAB-MA microspheres and EB/CAB-LP microspheres.

	Zero Order	First Order	Higuchi Equation
EB/CAB-MA	0.8181	0.9590	0.9132
EB/CAB-LP	0.9219	0.9837	0.9633
